# Psychological and Somatic challenges identified in a Menopause Rating Scale assessment of women living with HIV in Lagos, Nigeria

**DOI:** 10.21203/rs.3.rs-7032996/v1

**Published:** 2025-08-06

**Authors:** Azuka Patrick Okwuraiwe, Opeoluwa Shodipe, Mabel Uwandu, Chika Kingsley Onwuamah, Oliver Chukwujekwu Ezechi, Lisa Flowers

**Affiliations:** Nigerian Institute of Medical Research; Nigerian Institute of Medical Research; Nigerian Institute of Medical Research; Nigerian Institute of Medical Research; Nigerian Institute of Medical Research; Emory University School of Medicine

**Keywords:** Menopausal, Quality of life, HIV, Menopausal symptoms, MRS, Psychological, Somatic, Urogenital

## Abstract

**Background:**

Human immunodeficiency virus (HIV) infection remains a global health challenge disproportionately affecting women. Women living with HIV (WLWH) may experience earlier and more intense menopausal symptoms than others, which may influence their retention in therapy and treatment response. A standardized life quality assessment scale, the menopause rating scale (MRS), is a health-related quality-of-life tool for symptoms evaluation and is valuable in diagnosing menopause challenges. The objective was to assess the menopausal psychological and somatic challenges of WLWH on antiretroviral therapy using the MRS, and evaluate occurrence of menopausal symptoms, in comparison with women free from the infection.

**Methods:**

A cross-sectional descriptive study was adopted. Adult WLWH (n=344), 35 to 65 years (encompassing the pre-, peri- and post-menopausal stages) on ART for at least two years were surveyed using the MRS questionnaire (psychological, somatic and urogenital) including socio-demographic queries. A control group (n=90) composed of age-matched women were also assessed for the same. Frequency distribution and p-values were calculated using Epi info (version 7).

**Results:**

Among the 344 WLWH, 157 (45.6%), 12 (3.4%) and 175 (51%), were pre-, peri-, and postmenopausal, respectively. Median age of menopause onset was 48 (IQR 42-52) and 49.3 (IQR 45-55) years. Somatic and psychological challenges were more experienced by the WLWH, comprising joint/muscle complaints, sleeping disorders, anxiety, depression, irritability and exhaustion at rates of 59.4%, 29.2%, 27.4%, 26.5%, 23.5%, and 22.3% respectively. Controls had somewhat similar levels of challenges, at 52.2%, 31.1%, 27.7%, 23.3%, 21.1%, and 18.8% respectively. Participants with severe symptoms were 3.2%.

**Conclusions:**

WLWH had major psychological and somatic challenges indistinctive from uninfected women, which included joint/muscle complaints, depression and anxiety. Menopausal WLWHexperience unique challenges that require thoughtful solutions. To enhance clinical care, it is essential to prioritize these factors that improve their quality of life.

## Background

Menopause marks the end of the reproductive phase of a woman's life, when complete depletion of ovarian follicles leads to loss of ovarian production of sex hormones.^1^ The importance of studies conducted in a clinical setting for ageing women has grown in recent years, raising the interest in measuring the health-related quality of life and symptomatology. Both women and men undergo a decline in physical and mental capacities as they age. Women however, frequently experience symptoms such as intermittent sweating or hot flashes, cognitive impairment, difficulty concentrating, anxiety, depressive episodes, sleep disturbances, and issues with bones and joints.^2^ Developed in the early 1990s to address the absence of standardized scales for assessing the severity of ageing symptoms and their effect on health-related quality of life (HRQoL), the Menopause Rating Scale (MRS) tool was designed for this purpose.^3^ Initially completed by the treating physician, the MRS was redesigned to allow for easy completion by women themselves.^4^ Understanding the intersection of menopause and Human Immunodeficiency Virus (HIV) is crucial, as women living with HIV (WLWH) are increasingly reaching menopausal age and experiencing a distinct set of health challenges.

According to the Joint United Nations Programme on HIV/AIDS (UNAIDS), in 2023 approximately 39.9 million people worldwide are living with HIV, and sadly, around 630,000 people died from AIDS-related causes, of which 53% of them were women and girls. This high prevalence recorded is due to increased proportion of women getting infected.^5^ In addition, the average life expectancy of individuals living with HIV has increased due to successful management with antiretroviral therapy (ART).^6^ Given the fact that HIV has evolved into a chronic medical condition, co-morbidities and other ageing-related conditions, such as menopause in women, would be given more consideration in clinical HIV practice.^7^ Unfortunately, HIV is still increasingly feminized particularly in sub-Saharan Africa.^8^

Reports indicate that WLWH are likely to undergo menopause prematurely^9^, with more significant symptoms and different reproductive hormone profiles than women free from the virus.^10,11^ Possible reasons for this might be due to HIV infection itself and other socio-demographic factors such as smoking, drug abuse and hepatitis B and C co-infection.^12^ Andany and colleagues reported a median menopausal age of 48 years among WLWH in Canada, as compared to 51 years among the general population.^13^ Differences in pharmacokinetics and efficacy of antiretroviral drugs between men and women have been observed. Certain menopausal attributes share similarities with symptoms of HIV infection or adverse effects of HIV medication, including irregularities in the menstrual cycle, alterations in skin and hair, emotional fluctuations, or occurrences of night sweats.^14,15^ Menopausal WLWH face several concurrent factors that increase their susceptibility to metabolic issues, such as osteoporosis, hormonal imbalances and changes in lipid and glucose metabolism.^16,17^ The intersection of multiple symptoms such as hot flashes/night sweats, sleep disturbances, and depression, which are associated with both HIV and/or menopause, pose challenges for women and their healthcare providers in pinpointing the underlying cause and devising suitable management strategies.^18,19^ Obtaining an in-depth comprehension of the potential interplay between HIV and menopausal symptoms is crucial, and they impact disease progression and treatment as individuals age.^20^ Some WLWH struggle with the extra burden of menopause alongside managing their condition, leading to difficulties adhering to medication and attending clinical appointments which worsen health.^21^ The body's natural processes for breaking down female sex hormones, such as estrogen and progesterone, share common pathways with those used to metabolize certain antiretroviral drugs.^22,14^

It has been established that there are differences between men and women in terms of response to highly active antiretroviral therapy (HAART).^23,24,25^ Enhancing women's health is a major public health challenge in sub-Saharan Africa. This study aimed to identify the most common somatic, psychological, urogenital menopausal symptoms experienced by WLWH in Lagos, Nigeria.

## Methods

### Ethics and Participant Consent

The study received ethical clearance from the Institutional Review Board (IRB) of Nigerian Institute of Medical Research (NIMR) (IRB/22/0077). All participants provided written informed consent prior to participation, and were assured of voluntary participation, confidentiality, and data protection throughout and after the study.

### Study Design

This study employed a comparative cross-sectional design to investigate the relationship between menopausal symptoms and quality of life among women living with HIV compared to age-matched uninfected women pre- to post menopause.

### Study Population and Location

A total of 434 participants were recruited for this study. Those who came for regular appointment visits were approached. Voluntary participation was requested from those who met the study inclusion criteria. The sampling technique used was purposive sampling, based on inclusion criteria.

### Inclusion and exclusion criteria

Participants included in the study were WLWH, aged between 35 to 65 years (to encompass years prior to and after menopause onset), consented to partake in the study, on first line antiretroviral therapy (ART), composed of tenofovir disoproxil fumarate (TDF) and lamivudine (3TC), or emtricitabine (FTC) and efavirenz (EFV) 600 mg, once daily dosage) for at least two years. This drug combination is the current first line standard of care for persons living with HIV in Nigeria based on treatment guidelines.

Participants were excluded based on pregnancy, co-infection with hepatitis B or C viruses, or tuberculosis (TB), which may act as confounders to obscure the aim of the study, use of hormonal birth control or hormone replacement therapy (HRT) or with a prior history of hysterectomy and bilateral oophorectomy.

### Definition of variables

The women were classified into premenopausal, perimenopausal and postmenopausal groups.

**Premenopausal** if they experienced regular menstrual cycles.

**Perimenopausal** if they had missed menses in at least three cycles, but no more than 11 within the last 12 months.

**Postmenopausal**, also known as **menopausal**, if they had amenorrhea for 12 months or longer.

### Questionnaire administration

The Menopause Rating Scale II (MRS) is a standardized tool to assess menopausal symptoms. It has been widely used to assess and evaluate the presence and severity of menopause symptoms in populations worldwide. This tool has been employed across various populations, including women living with HIV.^26^ It was adapted from the PRIME study of Tariq et al in England.^27^ MRS is composed of three subscales, the somatic, psychological and urogenital sub-scales, with 4, 4 and 3 questions in each subscale respectively. Information regarding socio-demographic details, health, sexual history medical background and HIV-related data was also collected. It was an interviewer administered questionnaire.

Each item is rated on a scale of 0 (not present), 1 (mild), 2 (moderate), 3 (severe), or 4 (very severe). The MRS total score was derived by summing the scores obtained for each subscale. Somatic, psychological, and urogenital symptoms were deemed present if the total scores for the respective subscales equaled or exceeded 3, 2, and 1, respectively. The MRS has a scoring scale of between 0 (asymptomatic) and 44 (highest degree of complaints). The scale has undergone content, construct, reliability and cross-cultural validity in Nigeria.^28^

### Data analysis

Menopause symptom data was collected on the MRS questionnaire hard copies and entered in Microsoft Excel spreadsheet. Quality assurance processes, such as independent double entries, verified the accuracy of data entry. Frequency distribution was used for the socio-demographic data (marital status, education). *p*-values were calculated using Epi info (version 7). *p*-values less than or equal to 0.05 were considered statistically significant. Symptoms of night sweating, depression, irritation and sexual difficulties were compared between the premenopausal and postmenopausal groups. These groups were compared to understand at which stage issues were more frequently experienced.

## Results

### Participant socio-demographics and sexual characteristics

A total of 344 patients and 90 controls participated in the study, and the average age of the entire cohort was 47.1 ± 7.2 years. Among the WLWH there were more married women (59.7%) than widowed (22.7%). Most (48.1%) of the study population had a secondary school education. Among the controls tertiary education was observed more (63.3%) as shown in Table 1. Out of the 344 WLWH, 157 (48.4%) were premenopausal, 175 (52.9%) were postmenopausal, and 12 (3.6%) were perimenopausal, while among controls, 23 (25.6%) were premenopausal, 65 (72.2%) postmenopausal, and 2 (2.2%) were perimenopausal.

**Figure 1:**
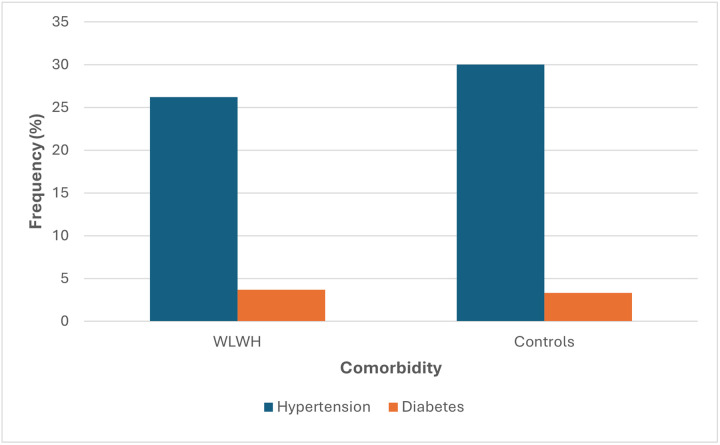
Self-reported chronic comorbidities of WLWH and controls

The premenopausal and postmenopausal groups had a mean age of 43 and 48 years, respectively. Mean age at menarche was 14.8 (range 10–21) and 13.9 (range 12–17) years for the WLWH and controls respectively. Among the post-menopausal women, the median age of menopause onset was 48 (range 42–52) years for WLWH and 49.3 (range 44–53) years for control group. Mean parity was 3 for both groups. Current and past heavy menstrual flows (menorrhagia) were reported by 38.8% and 43.3% of WLWH and controls respectively. Over half (54.3%) of all women, reported currently using a form of contraceptive (Table 1). Total lifetime sexual partners for both groups were a median of 2. Only 34 (10.1%) and 9 (10%) women had heard of or were aware of HRT among the WLWH and the controls respectively. Co-morbidities of hypertension and diabetes were 26.3%, 3.7%, and 30%, 3% for the WLWH and controls respectively (Fig. 1).

### Menopausal symptoms and severity based on the MRS tool

Among the WLWH the mean total MRS score was 4.1 ± 3.6, with a range of 0 to 16. More than half (206; 61.6%) had a total MRS score of 0–4, interpreted as no/minimal symptoms. There were 88 women (26.3%) with mild symptoms (score 5–8), 40 (11.9%) with moderate symptoms (score 9–15) and 11 (3.2%) had severe symptoms (score 16 and above). On the subscale level, mild somatic symptoms were prevalent across all menopausal stages at rates of 14.2%, 33.3%, and 14.3%, for the premenopausal, perimenopausal and postmenopausal respectively. Similarly, mild psychological symptoms were observed across the three stages. In contrast, urogenital symptoms, exhibited a notably lower prevalence compared to the other subscales. There was no statistically significant difference in the somatic, psychological and urogenital symptoms among those at the pre, peri and post-menopausal stages. Detailed somatic, psychological, and urogenital reports from the MRS are illustrated in Table 2.

In the somatic subscale, on both the WLWH and controls, the most prominent symptom was joint/muscle discomfort, affecting more than half of the participants (59.4% and 52.2%), followed by sleep disturbances (29.2% and 31.1%) and night sweats/hot flashes (25.3% and 28.8%). Depression, irritability, anxiety, and fatigue were relatively prevalent, each affecting approximately 20% or more of the cohort, as presented in Table 3. The predominant urogenital symptom reported was vaginal dryness, identified by 21.4% and 13.3% in WLWH and controls respectively.

The degree of symptom severity was assessed according to age group. In the three age groups, mild symptoms were reported equally (59%, 59.8% and 59.6% for 35–44, 45–54 and 55–65 years, respectively) for the WLWH. Severe symptoms were reported increasingly with age, as 10.9%, 11.9% and 14.6% for 35-44-, 45-54- and 55–65-year age groups, respectively, but the reverse occurred in the control group, as shown in Table 4.

## Discussion

The demography within Nigeria and sub-Saharan Africa is shifting due to a growing aging population and longer life expectancies, therefore sub-Saharan African women will spend a significant portion of their lives in post menopause. This study assessed the menopausal symptoms observed by WLWH of the pre- to post-menopausal age range in comparison with apparently healthy women. The occurrence of somatic, psychological and urogenital symptoms was reported through the pre-, peri- and postmenopausal stages. Utilizing the MRS, significant levels of somatic symptoms including sleeping disorders (lack of sleep, nightmares) cardiac complaints, and night sweating were found in both groups, with over half of the cohort experiencing joint and muscle pain. Similar symptoms of joint/muscle discomfort, physical and mental exhaustion and sexual problems were observed in a study of Zimbabwean women living with HIV.^29,30^ In addition, notable psychological symptoms were reported including depression, anxiety, irritation and exhaustion. Older age (55–65 years) of women was associated with increasing symptom severity (10.9–14.6%). However, these occurrences were not statistically significantly different between age groups of both the WLWH and the apparently healthy women.

The prevalence of co-morbidities among WLWH, hypertension (26.3%) and diabetes (3.7%), raises significant concerns, particularly in the context of HIV's increasing comorbidity with chronic noncommunicable diseases as the epidemic enters its fourth decade. However, this was not significantly different from the control group. Relating to joint and muscle complaints, a study on pain by menopausal women aged 45–60 years showed the importance of eliciting a history of pain and addressing symptoms to improve wellbeing.^31^

WLWH and HIV-uninfected women exhibited comparable symptoms across psychological, somatic, and urogenital domains. A Cambodian study on menopausal WLWH mirrors our findings, particularly in the high frequency of psychological symptoms such as physical and mental exhaustion, irritability, and depression.^32^ Similar results were observed in a study of menopause attitudes in 30- to 35-year-old women with or at risk of HIV in USA.^33^ HIV status did not influence their attitude towards menopause. In contrast to this present study, a 2005 study among blacks and Hispanics showed that HIV-infected women reported more menopause symptoms than HIV-uninfected women, but symptoms were less frequent in women with more advanced HIV disease.^34^ However, it differed from the present study due to having 30% illicit drug users and a majorly premenopausal (48%) cohort.

The occurrence of these symptomatic challenges identified in this study, demonstrate a need for crucial focus on this age strata of women. In the USA, the Women's Interagency HIV Study (WIHS) comprehensively monitored women extensively from 1993 to 2018 and documented the changes in mortality. It indicated that HIV infection affects women differently, with varying impacts on health outcomes, including disease progression, treatment response, and comorbidity risks, compared to men.^35^ Therefore it would be plausible to infer that HIV tends to affect menopausal women more.

Alarmingly about 21% of the study population consumed alcohol, despite its potential harm to individuals living with HIV. Such unhealthy habits, coupled with socioeconomic challenges like unemployment and food insecurity, may worsen menopausal symptoms, emphasizing the need for targeted education on the risks associated with alcohol use.

The study's findings indicate that WLWH experience similar menopausal symptoms as women without HIV. This similarity has significant implications for the care and treatment of women living with HIV, spanning clinical, public health, and research areas.

Clinically, the findings suggest that similar treatment approaches can be used for WLWH and those without HIV. Healthcare providers can focus on managing menopausal symptoms, such as hot flashes, vaginal dryness, and mood changes, rather than modifying treatment approaches based on HIV status. From a public health perspective, reducing stigma around HIV and menopause is crucial, emphasizing that women living with HIV experience similar menopausal symptoms as others. Increasing awareness among healthcare providers and the public is essential, recognizing that menopausal symptoms are a common experience for women living with HIV, warranting attention and care.

Public health initiatives can promote integrated care models, addressing both HIV and menopausal health, to improve overall well-being for WLWH. These findings require for partners and families to provide emotional support and understanding to women during menopause, encourage open communication about symptoms and concerns and assist with daily tasks to alleviate stress and exhaustion. Healthcare providers should conduct regular menopause-related health checks, screen for depression, anxiety, and other psychological symptoms, develop personalized treatment plans addressing all symptoms, and consider age-related symptom severity when managing menopause in women. They should seek medical attention for menopause-related symptoms, prioritize self-care, exercise, balanced diet, stress management and join support groups to share experiences. Lastly policy makers need to develop guidelines for menopause care, increase awareness on menopause and HIV while ensuring access to comprehensive healthcare services.

This study's methodology of self-reported questionnaire data has limitations. Participant responses may be susceptible to self-reporting biases, such as recall bias and social desirability bias, potentially compromising the accuracy of reported symptoms and behaviors. Furthermore, the study site, limited to Lagos, may not be representative of Nigeria's diverse population, which could impact the generalizability of the findings.

## Conclusions

This study underscores the significance of addressing psychological and somatic symptoms in menopausal women living with HIV. To enhance clinical care incorporating routine psychological and physical assessments are recommended into regular hospital visits, prioritizing the factors that improve their quality of life. WLWH had major psychological and somatic challenges indistinctive from uninfected women. Menopausal WLWH experience unique challenges that require thoughtful solutions.

## Figures and Tables

**Table 1:** Socio-demographics, lifestyle and sexual practices of WLWH and controls

Indices	WLWH Controls
	N % Range N % Range
Age at menarche (years)	14.8 - 10–21 13.9 - 12–17
Marital Status: Single	41 11.9 - 15 16.7 -
Married	197 57.3 - 60 66.6 -
Divorced	18 5.2 - - - -
Separated	12 3.5 - 9 10 -
Widowed	76 22.1 - 6 6.7 -
Education: No formal	10 2.9 - 5 5.6 -
Primary	41 11.9 - 7 7.8 -
Secondary	163 47.4 - 21 23.3 -
Tertiary	130 37.8 - 57 63.3 -
Median lifetime sexual partners	2 - 0–10 2 - 1–8
Median parity (living, dead)	3 - 0–7 3 - 0–8
Age at menopause (years)	48 - 44–56 49.3 - 45–55
Attained menopause	175 51 - 48 53.3 -
Menorrhagia (heavy flow)	130 37.7 - 39 43.3 -
Contraceptive use (all forms)	182 52.9 - 57 63.3 -
Sexually active	186 54 - 57 63.3 -
Alcohol use (varied levels)	71 20.6 - 18 20 -
Cigarette smoking	2 0.6 - 1 1.1 -

**Table 2:** Proportion of WLWH reporting no, mild, moderate, or severe somatic,psychological, and urogenital symptoms considered by menopausal status

Menopausal Status				
Symptom	Premenopausal	Perimenopausal	Postmenopausal	*p*-value
	N = 157	N = 12	N = 175	
Somatic N (%)				
None	121 (71.6)	7 (58.3)	131 (74.8)	< 0.001
Mild	23 (14.2)	4 (33.3)	25 (14.3)	
Moderate	14 (8.9)	1 (8.4)	14 (8.0)	
Severe	9 (5.3)	0 (0.0)	5 (2.9)	
Psychological N (%)				
None	114 (67.4)	7 (58.3)	138 (78.9)	< 0.001
Mild	35 (20.7)	3 (25)	23 (13.1)	
Moderate	15 (8.9)	2 (16.7)	12 (6.8)	
Severe	5 (3.0)	0 (0.0)	2 (1.1)	
Urogenital N (%)				
None	137 (86.2)	10 (83.3)	163 (93.1)	< 0.001
Mild	12 (7.5)	2 (16.7)	8 (4.7)	
Moderate	7 (4.4)	0 (0.0)	2 (1.1)	
Severe	3 (1.9)	0 (0.0)	2 (1.1)	

**Table 3:** Frequency of reported individual somatic, psychological, and urogenital symptoms among WLWH and apparently healthy controls

Symptom	WLWH N (%)	Controls *p* value N (%)
Somatic		
sweating/hot flashes	85 (25.3)	26 (28.8)
cardiac complaint	79 (23.5)	21 (23.3) 0.930
sleeping disorders	98 (29.2)	28 (31.1)
joint/muscle complaints	199 (59.4)	47 (52.2)
Psychological		
depression	89 (26.5)	21 (23.3) 0.365
irritability	79 (23.5)	19 (21.1)
anxiety	92 (27.4)	25 (27.7)
exhaustion	75 (22.3)	17 (18.8)
Urogenital		
sexual difficulties	36 (10.7)	6 (6.6) 0.525
urinary challenges	18 (5.3)	6 (6.6)
vaginal dryness	72 (21.4)	12 (13.3)

**Table 4:** Frequency of MRS symptom severity reports by age group

Age group (years)	N	WLWH Symptom severity *p* value		
		Mild N (%)	Moderate N (%)	Severe N (%) 0.99
35–44	100	152 (59)	75 (29.4)	28 (10.9)
45–54	190	257 (59.8)	69 (28.3)	29 (11.9)
55–65	54	53 (59.6)	23 (25.8)	13 (14.6)
	N	Controls Symptom severity p value		
		Mild N (%)	Moderate N (%)	Severe N (%) 0.99
35–44	20	48 (46)	33 (32)	28 (22)
45–54	45	89 (52)	25 (38)	13 (10)
55–65	25	35 (56)	11 (36)	7 (8)

## Data Availability

Data is provided within the manuscript and in a supplementary information file.

## List of Abbreviations

HIV: Human Immunodeficiency Virus
MRS: Menopause Rating Scale
HLQoL: Health-Related Quality of Life
WLWH: Women Living With HIV
ART: Antiretroviral Therapy
NIMR: Nigerian Institute of Medical Research
IRB: Institutional Review Board
WIHS: Women's Interagency HIV Study
